# Derivation and comparison of formulae for the adjustment of total calcium

**DOI:** 10.3389/fendo.2023.1070443

**Published:** 2023-05-12

**Authors:** Maria Phylactou, Alexander N. Comninos, Ahmed Salih, Marina Labib, Pei Chia Eng, Sophie A. Clarke, Pope Moore, Tricia Tan, Jaimini Cegla, Waljit S. Dhillo, Ali Abbara

**Affiliations:** ^1^ Section of Endocrinology and Investigative Medicine, Imperial College London, London, United Kingdom; ^2^ Department of Endocrinology, Imperial College Healthcare National Health Service Trust, London, United Kingdom; ^3^ Department of Clinical Biochemistry, North West London Pathology, Imperial College Healthcare National Health Service Trust, London, United Kingdom

**Keywords:** calcium, albumin, adjustment calcium equation, vitamin D, parathyroid hormone

## Abstract

**Background:**

Free ionized calcium (Ca^2+^) is the biologically active component of total calcium (TCa) and hence responsible for its biological action. TCa is routinely adjusted for albumin using several formulae (e.g. James, Orell, Payne and Berry) to more closely reflect Ca^2+^. Here, we derive a novel formula to estimate Ca^2+^ and compare its performance to established formulae.

**Methods:**

*Cohort for prediction of Ca^2+^*: 2806 serum samples (TCa) taken contemporaneously with blood gas samples (Ca^2+^) at Imperial College Healthcare NHS Trust were used to derive formulae to estimate Ca^2+^ using multivariable linear regression. *Cohort for prediction of PTH:* Performance of novel and existing formulae to predict PTH in 5510 patients was determined by Spearman correlation.

**Results:**

*Ca^2+^ prediction Cohort*: Adjusted calcium (r^2^ = 0.269) was less strongly associated with Ca^2+^, than TCa (r^2^ = 0.314). Prediction of Ca^2+^ from a newly derived formula incorporating TCa, potassium, albumin, and hematocrit had an improved r^2^ of 0.327, whereas inclusion of all available parameters increased the r^2^ further to 0.364. Of the established formulae, James performed best in predicting Ca^2+^ (r^2^ = 0.27). *PTH prediction cohort:* Berry resulted in higher whereas Orell in lower adjusted calcium levels. Prediction of PTH was strongest in the setting of hypercalcemia, with James having the highest Spearman correlation coefficient (+0.496) similar to including all parameters (+0.499).

**Conclusion:**

Adjustment of calcium for albumin using established formulae does not always outperform unadjusted TCa in the reflection of Ca^2+^. Further prospective studies are needed to optimise adjustment of TCa and to establish bounds for validity.

## Introduction

Calcium is a ubiquitous mineral that has a critical role across several physiological processes (1, 2). Intracellular calcium is involved in action potential regulation, cell signalling, glycogen metabolism, and hormone secretion (1, 2). Extracellular calcium plays a vital role in the coagulation cascade, maintenance of membrane integrity and muscle contraction (2, 3). Only 1% of total body calcium is present within body tissues or extracellular fluid, whereas the majority resides in bone with hydroxyapatite crystals acting as a calcium reservoir (3, 4).

Over a century ago, Rona and Takashi discovered that total serum calcium (TCa) separates into diffusible (~55%) and non-diffusible fractions (~45%) (5, 6), depending on pH and temperature (7). Of the non-diffusible calcium, ~81% is albumin-bound and ~19% is globulin-bound (7). The majority (~80%) of the diffusible calcium portion is free ionized calcium (Ca^2+^) (7) and the remainder (5-15%) is complexed with small anions such as bicarbonate, citrate and phosphate (8). McLean and Hastings provided evidence that Ca^2+^ was the biologically active component (9, 10), with its concentration predominantly dictated by parathyroid hormone (PTH) and vitamin D (11). Crucially, several studies have demonstrated that changes in Ca^2+^, rather than TCa, most strongly regulate PTH secretion (12–14). Thus, estimation of Ca^2+^ is paramount for correct interpretation of PTH levels when assessing patients with disorders of calcium homeostasis. This can be particularly important when patients have borderline calcium levels, whereby the interpretation of PTH levels can diverge between being an appropriate physiological response, to being inappropriate for that calcium status and thus consistent with parathyroid disorders.

However, measuring Ca^2+^ concentrations requires direct ion selective electrodes (ISE) that are not universally available in the biochemistry laboratory (15). TCa concentration can vary according to albumin concentration (16), however the ionized calcium fraction (Ca^2+^) remains unaltered (17). Consequently, calcium is usually adjusted for the level of albumin with the aim of deriving a closer representation of Ca^2+^. The first formula used to adjust TCa for albumin was described by Payne and colleagues in 1973 (18). Since then several formulae have been derived and compared to this formula (19–21), however whether the accuracy of some of these formulae is better than the unadjusted TCa has been questioned (21–26).

This study aims to assess whether routinely measured analytes and baseline factors can be used to better predict Ca^2+^ and assess how this compares with established formulae and unadjusted TCa. We audited samples analysed during routine clinical practice from patients who had contemporaneous measurement of both Ca^2+^ (using blood gas analyser ISEs) and TCa (using automated lab analyser) to evaluate the effect of parameters including gender, age, creatinine, total protein, albumin, globulin, phosphate, sodium, potassium, creatinine, hemoglobin, and hematocrit, that could affect prediction of Ca^2+^ from TCa. We also identified boundaries for these parameters beyond which the relationship between TCa and Ca^2+^ was lost e.g., extremes of albumin.

Thereafter, having evaluated the performance of existing formulae to reflect Ca^2+^ and having derived a novel formula to predict Ca^2+^, we then evaluated the performance of this formula (as well as other available established formulae for adjustment of TCa for albumin) to assess their ability to predict PTH in samples from a second cohort of patients with known vitamin D and PTH levels.

## Materials and methods

This work was conducted as part of a service evaluation of calcium estimation at Imperial College Healthcare NHS Trust (ICHT), reference number: ASM_044.

### Study cohorts

Data were obtained from two cohorts, with the first cohort (Ca^2+^ Prediction Cohort) being used to derive a novel formula to predict Ca^2+^ from TCa (and compare its performance to that of established formulae that adjust for albumin), and the second (PTH Prediction Cohort) being used to evaluate the performance of the newly derived formula (as well as established formulae) in the prediction of PTH. Data for both cohorts were extracted from laboratory data for the period 1 June 2016 to 29 June 2019.

For the first cohort (Ca^2+^ Prediction Cohort), we collated data from 2806 patients who had contemporaneous collection of serum samples (providing TCa) and blood gas samples (providing Ca^2+^) in order to estimate the relationship between Ca^2+^ and TCa. As blood gas analysis was usually conducted in the acute setting, we also repeated the analysis in a restricted subset of this cohort of 537 patients with complete data, who had normal pH (7.35-7.45), creatinine (<120 μmol/L), albumin (>15 g/L), total protein (>40 g/L) and calcium (2.2-2.6 mmol/L) levels to exclude those with markedly perturbed results due to significant ill health. TCa, adjusted calcium (as reported by the ICHT pathology lab based on the formula: TCa + 0.013*(40 – Alb) which was derived by J. Meek from internal data using Abbott bromocresol-purple (BCP) albumin assay & calcium method), creatinine, albumin, globulin, phosphate, total protein, sodium, potassium, hemoglobin, and hematocrit were measured in the clinical chemistry lab. Although gender, and age, were available, data on individual patient diagnoses/medications were not available. The relationship between Ca^2+^ and these other parameters was determined from this cohort using multivariable linear regression.

The second cohort (PTH Prediction Cohort) comprised of 5510 patients with the same laboratory measures available as in the derivation cohort, but with the addition of PTH and vitamin D levels. This second cohort was more commonly outpatient based as vitamin D and PTH are not usually measured acutely, whereas Ca^2+^ was not available in this cohort as blood gas analysis is not usually conducted in this setting. Thus, the ability of Ca^2+^ predicted by the formulae from the first cohort to predict PTH was assessed in this second cohort using Spearman correlation analysis.

### Assays

TCa (Arsenazo III method), creatinine (Jaffe method), albumin (Bromo-cresol purple method), phosphate (phosphomolybdate UV method), total protein (Biuret method), serum sodium (Indirect ISE method), and serum potassium (Indirect ISE method) were measured on the Architect 2000 platform (Abbott, Maidenhead, UK). Haematocrit and haemoglobin were measured using Sysmex XE2100 analysers at Imperial College Healthcare NHS Trust hospitals. Ca^2+^ was measured on the GEM 2000 blood gas analyser (Werfen, Warrington, UK). The intra- and inter-assay coefficients of variation for all assays were <5% within the reportable range.

### Statistical analysis

Analysis was performed using Prism v9.3 (GraphPad), and Stata v13.0 (StataCorp). Evidence for non-parametric distribution was assessed using the D’Agostino-Pearson test. Data are presented as mean ± standard deviation ( ± SD) for parametrically distributed data, or median (interquartile range; IQR) for non-parametrically distributed data. Statistical comparison across multiple groups was performed using one-way ANOVA with *post hoc* Tukey’s multiple comparison test, or Kruskal-Wallis test with *post hoc* Dunn’s test, as appropriate. Multivariable linear regression was used for prediction of Ca^2+^. Spearman correlation was used to assess correlations between calcium and PTH stratified by vitamin D category and calcium below and above 2.5 mmol/L.

## Results

Baseline characteristics of both the first and second cohorts are summarized in **Table 1**, including both the unrestricted datasets, as well as restricted datasets (i.e., only those with pH 7.35-7.45, creatinine <120 μmol/L, albumin >15 g/L, total protein >40 g/L and calcium 2.2-2.6 mmol/L).

**Table 1:** Basic characteristics of the patients in the Ca^2+^ Prediction cohort (on the left) and the PTH Prediction cohort (on the right).

	COHORT TO PREDICT IONIZED CALCIUM		COHORT TO PREDICT PTH		
	TOTAL COHORT	RESTRICTED COHORT	TOTAL COHORT		RESTRICTED COHORT
**Patients**	2806	537	5510		2659
**Male**	1487 (53%)	258 (%)	1832 (33.25%)		815 (30.65%)
**Female**	1154 (41%)	279 (%)	3678 (66.75%)		1844 (69.35%)
**No gender assigned**	165 (6%)	N/A	N/A		N/A
**Age (years)**	59 (36, 76)	55 (35,73)	57 (44,69)		53 (41, 65)
**Total calcium (mmol/l)**	2.24 (2.06, 2.38)	2.34 (2.28,2.42)	2.39 (2.30, 2.48)		2.38 (2.31, 2.46)
**Ionised calcium (mmol/l)**	1.13 (1.05, 1.19)	1.17 (1.13, 1.21)	N/A		N/A
**Adjusted calcium (mmol/l)**	2.31 (2.19, 2.42)	2.38 (2.32, 2.45)	2.40 (2.33, 2.50)		2.39 (2.33, 2.47)
**Globulin (g/l)**	31 (26,35)	33 (30, 37)	34 (30, 37)		33 (30, 37)
**Total protein (g/l)**	65 (57, 72)	71 (66, 75)	73 (69, 77)		73 (70, 76)
**Phosphate (mmol/l)**	1.13 (0.93, 1.39)	1.07 (0.9, 1.20)	1.08 (0.95, 1.22)		1.1 (0.97, 1.22)
**PTH**	N/A	N/A	7.4 (5.1, 11.5)		7.2 (5.3, 10.3)
**Vitamin D**	N/A	N/A	64.6 (44, 84.8)		61.1 (41.1, 81.6)
**Albumin (g/l)**	34 (28, 38)	38 (35, 40.5)	39 (37, 41)		39 (37, 42)
**Serum Na (mmol/l)**	138 (136, 140)	139 (136, 140)	140 (138, 141)		140 (139, 141)
**Serum K (mmol/l)**	4.2 (3.9, 4.6)	4.2 (3.9, 4.4)	4.3 (4.1, 4.6)		4.3 (4.1, 4.6)
**Creatinine (μmol/l)**	76 (63, 106)	72 (63,83)	72 (63, 90)		69 (62, 79)
**Hemoglobin (g/l)**	129 (114, 144)	136 (124, 148)	131 (120, 142)		133 (123, 143)
**Hematocrit (ratio)**	0.39 (0.35, 0.43)	0.41(0.37, 0.44)	0.40 (0.37, 0.43)		0.41 (0.38, 0.44)

The restricted cohorts include only samples from adult patients with normal pH (7.35-7.45), normal creatinine (<120 μmol/L), albumin (>15 g/L) and total protein (>40 g/L) levels. Numbers represent median with interquartile range values for continuous data, or number of patients with percentages for categorical data.

### Prediction of Ca^2+^from TCa in cohort 1

We firstly assessed the boundaries for which the relationship between *TCa* and *Ca^2+^* was altered for available biochemical variables. The relationship was altered in those with pH values <7.24, albumin concentrations ≤15 g/L, total protein <40 g/L, and creatinine >120 μmol/L (**Supplemental Figure 1**). To further assess the relationship between TCa and Ca^2+^ in a cohort not affected by the above extreme values, we restricted the cohort to adults with pH 7.35-7.45, creatinine <120 μmol/L, globulin >15 g/L, albumin >15 g/L, total protein >40 g/L and TCa within the reference range (2.2-2.6 mmol/L).

**Figure 1:**
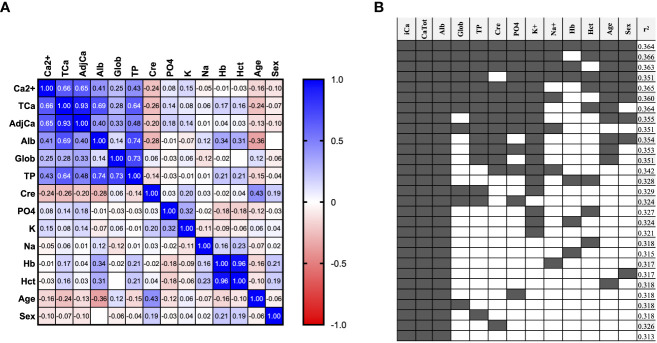
Prediction of Ca^2+^ from routinely available variables. **(A)** Correlation matrix showing Spearman correlation coefficient of all available variables in the unrestricted Ca^2+^ Prediction Cohort (n=2806 samples). **(B)** Multivariable linear regression analysis of different combinations of variables for the prediction of ionized calcium in the unrestricted Ca^2+^ Prediction Cohort. (n=2806 samples). F1: (TCa*.33079) + (alb*.0122872) + (age*-.0001313) + (glob*.0131042) + (TP*-.0116891) + (Phos*-.0186232) + (Na*-.000539) + (K*.014544) + (Cre*-.0001297) + (Hb*.0004036) + (Hct*-.3759772) + (MaleSex*-.011839) + 0.4634889. F2: (TCa*.3368484) + (alb*.0016509) + (K*.0001544) + (Hct*-.2152976) + 0.3835567.

A correlation matrix was used to present univariable Spearman Correlation Coefficients for available parameters in both the unrestricted (**Figure 1A**) and restricted cohorts (**Supplemental Figure 2A**). In the unrestricted cohort, Ca^2+^ correlated similarly with TCa (r=0.66) compared to adjusted calcium by the local adjustment formula (J.Meek) (r=0.65) (**Figure 1A**). However, in the res*tricted cohort* (**Supplemental Figure 2A**)*, a stronger correlation was observed between* Ca^2+^ *and adjusted calcium (r=0.40) than with TCa (r=0.36).*

We then performed multivariable linear regression to identify which combination of variables best predicted Ca^2+^ (**Figure 1B**, **Supplemental Figure 2B**). Using all twelve variables provided near the highest r^2^ of 0.364 in the unrestricted (**Figure 1B**), and 0.195 in the restricted cohort (**Supplemental Figure 2B**). This formula including all 12 routinely available biochemical parameters will be referred to as ‘Formula 1’ or F1 in subsequent analyses. We also identified a formula using a more pragmatic selection of just four variables that maintain similar performance: TCa, Albumin, K and Hematocrit (Hct) for unrestricted cohort r^2^ 0.327 (**Figure 1B**) and for restricted cohort r^2^ 0.172 (**Supplemental Figure 2B**), which will be referred to as ‘Formula 2’ or F2 in subsequent analyses.

We then performed multivariable linear regression to predict Ca^2+^ with predictors including TCa (or adjusted calcium for that analysis) and each other variable in turn (**Table 2**). In the unrestricted cohort (**Table 2**, left-sided panel), the r^2^ for the prediction of Ca^2+^ from TCa using our new formula F2 was 0.327, and was higher than using calcium adjusted for albumin by any of the established formulae (r^2^ 0.133 - 0.271). This r^2^ of our new formula F2 was only marginally increased by the addition of any other single variable to a maximum r^2^ of 0.326. In the restricted cohort (**Table 2**, right-sided panel), the r^2^ for TCa vs Ca^2+^ was lower at 0.099. Of the established albumin adjustment formulae, the James formula performed best with an r^2^ of 0.109 compared to 0.058 - 0.086 for other established formulae.

**Table 2:** Multivariable linear regression analysis of all variables with TCa vs Ca^2+^ and the four albumin-adjusted formulae vs Ca^2+,^ in the Ca^2+^ Prediction cohort (total cohort on the left and restricted cohort on the right).

VARIABLE	UNRESTRICTED COHORT			RESTRICTED COHORT		
	R^2^	p-value	Equation	R^2^	p-value	Equation
**Total Calcium (mmol/l)**	0.314	<0.001	=(TCa*0.36) + 0.31	0.099	<0.001	=(TCa*0.23) + 0.63
**Adjusted Calcium (mmol/l)**	0.269	<0.001	=(AdjCa*0.41) + 0.15	0.106	<0.001	=(AdjCa*0.25) + 0.57
**Albumin (g/l)​**	0.313	<0.001	=(Alb*0.0005) + (TC*0.35) + 0.32	0.116	<0.001	=(Alb*-0.002) + (TC*0.27) + 0.60
**Globulin (g/l)​**	0.318	<0.001	=(Glob*0.001) + (TC*0.35) +0.29	0.100	<0.001	=(Glob*0.0004) + (TC*0.22) + 0.62
**Total Protein (g/l)**	0.317	<0.001	=(TP*0.001) + (TC*0.32) + 0.31	0.100	<0.001	=(TP*-0.0008) + (TC*0.25) +0.63
**Phosphate (mmol/l)**	0.319	<0.001	=(PO4*-0.22) +(TC*0.36) + 0.33	0.099	<0.001	=(PO4*-0.005) + (TC*0.23) + 0.64
**Serum Na (mmol/l)**	0.317	<0.001	=(Na*-0.001) + (TC*0.36) + 0.51	0.109	<0.001	=(Na*-0.0018) + (TC*0.23) + 0.85
**Serum K (mmol/l)​**	0.319	<0.001	=(K*-0.0001) + (TC*0.36) + 0.30	0.130	<0.001	=(K*0.28) + (TC*0.21) + 0.55
**Creatinine (μmol/l)​**	0.326	<0.001	=(Cre*-0.0001) + (TC*0.34) + 0.35	0.099	<0.001	=(Cre*0.000024) + (TC*0.23) + 0.63
**Hemoglobin (g/l)**	0.314	<0.001	=(Hb*-0.0004) + (TC*0.36) + 0.35	0.140	<0.001	=(Hb*-0.0007) + (TC*0.25) + 0.67
**Hematocrit (ratio)**	0.316	<0.001	=(Hct*-0.18) + (TC*0.36) + 0.37	0.140	<0.001	=(Hct*-0.26) + (TC*0.25) + 0.68
**Age (years)**	0.319	<0.001	=(Age*-0.00004) + (TC*0.36) + 0.31	0.099	<0.001	=(Age*-0.000019) + (TC*0.23) + 0.63
**Gender**	0.317	<0.001	=(Sex*-0.018 if male) + (TC*0.35) + 0.33	0.110	<0.001	=(Sex*-0.013 if male) + (TC*0.23) + 0.63
**James Formula (mmol/l)** Total Calcium + [0.012*(39.9-Alb)]	0.271	<0.001	=(JamesCa*0.41) + 0.16	0.109	<0.001	=(JamesCa*0.25) + 0.56
**Payne Formula (mmol/l)** Total Calcium + [0.0246*(40.4-Alb)]	0.133	<0.001	=(PayneCa*0.31) + 0.37	0.058	<0.001	=(PayneCa*0.14) + 0.83
**Orell Formula (mmol/l)** Total Calcium + [0.0176*(34-Alb)]	0.218	<0.001	=(OrellCa*0.39) + 0.23	0.086	<0.001	=(OrellCa*0.20) + 0.69
**Berry Formula (mmol/l)** Total Calcium + [0.0227*(46-Alb)]	0.156	<0.001	=(BerryCa*0.33) + 0.27	0.065	<0.001	=(BerryCa*0.16) + 0.77
**Formula 1 (all variables)**	0.364	<0.001	=(TC*0.33) + (Alb*0.012) + (Glob*0.013) +(TP*-0.012)+(P04*-0.019)+ (Na*0.0005) + (K*0.15) + (Cre*-0.0001) + (Hb*0.0004) + (Hct*-0.38) + (Age*-0.0001) +(Sex*-0.012 if male) + 0.46	0.195	<0.001	=(TC*0.26) + (Alb*0.021) + (Glob*0.022) + (TP*-0.023) + (PO4*-0.022) + (Na *0.0007) + (K*0.029) + (Cre*0.0002) + (Hb*-0.0001) + (Hct*-0.18) + (Age*-0.0004) + (Sex*-0.012 if male) + 0.69
**Formula 2 (F2) TCa, Alb, K and Hct**	0.327	<0.001	=(TC*0.34) + (Alb*0.002) + (K*0.0002) + (HCT*-0.22) +0.38	0.172	<0.001	=(TC*0.25) + (Alb*-0.0007) + (K*0.026) + (Hct*-0.23) + 0.59

### Prediction of PTH using different calcium adjustment formulae in Cohort 2 (PTH Prediction Cohort)

**Figure 2:**
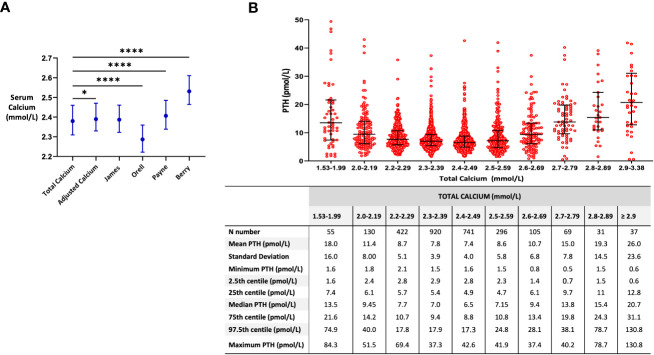
Associations between formulae for calcium adjustment and serum PTH levels. **(A)** Median (IQR) of calcium by different formulae for calcium adjustment. Comparison made by the Friedman test * p=0.0193, **** p=<0.0001. **(B)** Median (IQR) of serum PTH levels across different total calcium categories. Eight data points (outliers) with PTH levels >60 pmol/L are not shown. Samples with total calcium levels <2.2 mmol/L and corresponding PTH levels <1.6 pmol/L (n=7) were excluded (suspected primary hypoparathyroidism).

We then aimed to assess whether PTH could be better predicted using either our newly derived formulae (F1 or F2) or established formulae for the adjustment of TCa for albumin. Baseline characteristics for this cohort are presented in **Table 1** (right-sided panel). The median (IQR) TCa for this cohort was 2.38 (2.31, 2.46) (**Figure 2A**). Adjustment of calcium for albumin by Berry resulted in a higher result 2.53 (2.46, 2.61), whereas by Orell resulted in a lower result 2.29 (2.22, 2.36) (**Figure 2A**).

In order to interpret PTH levels appropriately, it is necessary to accurately categorize patients according to their calcium status i.e. as either being normocalcemic or hypercalcemic. This is crucial for correct clinical interpretation and subsequent management. To assess the impact of different adjustments of calcium for albumin on the categorization of patients as normocalcemic or hypercalcemic, we selected patients with borderline TCa levels (i.e. 2.59-2.61 mmol/L) and assessed the proportion of patients that would be classified as hypercalcemic (>2.6 mmol/L) using each of the albumin-adjustment calcium formulae. The proportion of these borderline cases (n=58) that would be categorized as hypercalcemic was: 22.4% for TCa, 29.3% if using ICHT adjusted Calcium formula, 29.3% by James formula, 1.7% by Orell formula, 31.0% by Payne and 81.0% by Berry. If we select patients with TCa levels between 2.55-2.65 mmol/L (n=109), then the proportion of patients that would be classified as hypercalcemic (>2.6 mmol/L) was: 31.5% for TCa, 43.5% if using ICHT adjusted Calcium formula, 45.4% by James formula, 13.9% by Orell formula, 51.9% by Payne, and 84.3% by Berry.

In order to better understand PTH ranges at different calcium levels, PTH values were plotted according to categories of TCa level. PTH levels had a U-shaped relationship with TCa levels, with PTH levels reaching a nadir at a TCa of between 2.40-2.49 mmol/L (**Figure 2B**). PTH values were higher in those with lower calcium levels (especially at TC <2.2 nmol/L) consistent with appropriate secondary hyperparathyroidism, and higher in those with higher calcium levels (TCa >2.5 nmol/L) consistent with primary hyperparathyroidism (**Figure 2B**).

Therefore, the relationship between calcium and PTH is non-linear, and thus we used Spearman correlation to assess the relationship between PTH and calcium measures, stratified by Vitamin D levels, and according to TCa categories (< 2.2 mmol/L, 2.2-2.49 mmol/L and ≥ 2.5 mmol/L) (**Table 3**). These showed that there is only a weak negative correlation between PTH and calcium measures in patients with a calcium level <2.5 mmol/L. The highest negative correlation (-0.478) was observed using the James formula in hypocalcemic patients with a TCa <2.2 mmol/L and a vitamin D level <20 nmol/L. Generally, the relationship between PTH and calcium was stronger in the setting of hypercalcemia (≥ 2.5 mmol/L) using most of the formulae regardless of vitamin D level. However, the James formula also provided the highest positive correlation coefficient between calcium and PTH in hypercalcemic patients (+0.496) which approached the newly derived F1 formula (using all available variables) (+0.499).

**Table 3:** Spearman Correlation analysis of the relationship between PTH and calcium measures from derived by the novel formulae (F1 and F2) and established formulae, stratified by Vitamin D levels, according to total calcium categories.

Vitamin D (nmol/L)	n	Total calcium	Adjusted calcium	James	Orell	Payne	Berry	F1	F2
				**Calcium < 2.2**					
**≤20**	36	-0.236	-0.433	-0.478	-0.463	-0.409	-0.409	-0.214	-0.086
**20.1-40**	43	-0.320	-0.330	-0.393	-0.348	-0.307	-0.307	-0.222	-0.219
**40.1-60**	43	0.071	0.164	0.082	0.140	-0.190	0.190	0.146	0.103
**60.1-80**	35	-0.403	-0.449	-0.372	-0.448	-0.438	-0.438	-0.483	-0.446
**80.1-100**	12	0.482	0.354	0.399	0.406	0.301	0.301	0.308	0.245
**100.1-120**	12	0.246	-0.067	-0.063	-0.084	-0.070	-0.070	-0.133	0.098
**>120**	4	-0.400	0.400	0.400	0.400	0.800	0.800	0.88	0.400
**<70**	**134**	**-0.208**	**-0.211**	**-0.272**	**-0.236**	**-0.186**	**-0.186**	**-0.135**	**-0.134**
**≥70**	**51**	**-0.044**	**-0.060**	**-0.110**	**-0.075**	**-0.043**	**-0.043**	**-0.095**	**-0.075**
				**Calcium 2.2-2.49**					
**≤20**	112	-0.153	0.076	-0.169	-0.140	-0.111	-0.117	-0.086	-0.134
**20.1-40**	362	-0.132	-0.002	-0.006	0.051	0.111	0.099	-0.054	-0.130
**40.1-60**	506	-0.066	-0.068	-0.035	-0.014	0.017	0.009	0.003	-0.033
**60.1-80**	533	-0.173	0.005	-0.129	-0.087	-0.039	-0.050	-0.105	-0.150
**80.1-100**	343	-0.043	0.010	-0.018	-0.002	0.014	0.010	-0.030	-0.026
**100.1-0120**	158	-0.151	0.046	-0.057	-0.006	0.032	0.026	-0.089	-0.144
**>120**	69	-0.113	-0.014	-0.015	0.029	0.067	0.067	0.086	-0.015
**<70**	**1243**	**-0.123**	**-0.014**	**-0.067**	**-0.029**	**0.016**	**0.004**	**-0.056**	**-0.109**
**≥70**	**840**	**-0.115**	**0.076**	**-0.061**	**-0.027**	**0.008**	**0.001**	**-0.047**	**-0.088**
				**Calcium >2.5**					
**≤20**	35	0.453	0.477	0.478	0.493	0.477	0.479	0.389	0.422
**20.1-40**	82	0.430	0.398	0.411	0.404	0.382	0.391	0.420	0.416
**40.1-60**	152	0.500	0.512	0.527	0.515	0.501	0.505	0.423	0.496
**60.1-80**	133	0.481	0.496	0.494	0.500	0.490	0.493	0.493	0.431
**80.1-100**	71	0.434	0.471	0.471	0.476	0.468	0.464	0.440	0.387
**100.1-120**	51	0.385	0.353	0.365	0.357	0.337	0.339	0.513	0.483
**>120**	14	0.780	0.665	0.697	0.670	0.657	0.657	0.394	0.643
**<70**	**339**	**0.474**	**0.462**	**0.481**	**0.467**	**0.451**	**0.455**	**0.431**	**0.462**
**≥70**	**199**	**0.446**	**0.493**	**0.496**	**0.495**	**0.490**	**0.489**	**0.499**	**0.415**

## Discussion

Ca^2+^ is considered the gold standard representation of biological calcium activity. However, measurement of Ca^2+^ is not practical, as most commercial vendors do not currently offer Ca^2+^ assay on large-scale automated instruments (27). Furthermore, even if Ca^2+^ assays were available on automated assay lines, most of these involve uncapping the tubes after centrifuging, which risks changing the pH of the sample and thus the Ca^2+^ concentration (27). Direct measurement of Ca^2+^ in clinical practice is most frequently conducted using blood gas analyser ISEs, which are not usually available in the outpatient setting. However, Ca^2+^ is more closely related to PTH levels than TCa and thus, a reliable formula for the estimation of Ca^2+/^adjustment of TCa from routinely measured biochemical parameters would aid in the interpretation of PTH levels. In particular, estimation of Ca^2+^ levels would be helpful in the interpretation of PTH in patients with borderline calcium results, whereby the classification of PTH can diverge between being an appropriate physiological response vs a pathological indicator of parathyroid disorders (28). In the present study, we found that the proportion of patients with borderline TCa levels (between 2.59-261 mmol/L) that would be classified as hypercalcemic varied hugely between 13.9 to 84.3% depending on the formula used for calcium adjustment, with a corresponding impact on the interpretation of PTH levels. This wide variation would therefore potentially result in different clinical management which may be ‘incorrect’ in some cases.

Interpretation of PTH levels is further challenged by the increasing recognition of normocalcemic hyperparathyroidism, whereby persistently raised PTH, which is unexplained by other abnormalities e.g. vitamin D < 50 nmol/L, could indicate parathyroid disease even without serum calcium levels becoming raised (28). In our data, PTH levels had a nadir at TCa levels between 2.4-2.49 mmol/L, although more typically endocrinologists use the upper limit of the reference range for TCa (~ 2.6 nmol/L) to interpret the appropriateness of that PTH level, which could have included patients with subclinical hyperparathyroidism in its derivation. Identifying the normal relationship between PTH and calcium is further complicated by the fact that patients have an individual set-point for calcium around which PTH secretion is altered (29), which is set at different levels on an individual basis within the reference range. Indeed, the relationship between calcium and PTH was strongest in the setting of hypercalcemia, with greater PTH levels begin associated with a greater degree of perturbation in calcium. Generally, PTH levels are used to distinguish hypercalcemia due to malignancy (physiologically suppressed) from that due to primary hyperparathyroidism (pathologically raised) (30). The lower limit of the reference range for PTH is typically used by most endocrinologists to distinguish these two conditions, however the rise in PTH levels in primary hyperparathyroidism especially in patients with higher calcium levels suggests that a higher threshold for PTH could be used to discriminate these two conditions, as otherwise a PTH level very close to the assay detection limit would be used. Furthermore, it is necessary to exclude renal calcium loss as an additional drive of increased PTH secretion (as a form of secondary hyperparathyroidism). Indeed, those labelled as being normocalcemic using TCa can in some instances have abnormal calcium when Ca^2+^ is assessed. Consistent with this, Koumakis *et. al.*, found that only 16 of 39 patients with normocalcemic (by TCa) hyperparathyroidism had both normal TCa and Ca^2+^ levels, further demonstrating the benefits of Ca^2+^ assessment to clarify diagnosis (31).

Notably, in the present study, we found that TCa outperformed adjusted calcium in the prediction of Ca^2+^ when including all the patients (unrestricted cohort) similar to previous studies (25, 32). We also describe parameters around which the relationship between TCa and Ca^2+^ was altered e.g. extremes of albumin level. It is possible that the relationship between TCa and Ca^2+^ is altered in patients who are acutely unwell, with both low and high Ca^2+^ being associated with increased mortality (33, 34) and Ca^2+^ also being proposed as an indicator of severity of COVID-19 (35). However, adjusted calcium formulae outperformed TCa in the restricted cohort, whereby extreme values for albumin, creatinine etc were excluded, perhaps reflecting that many of these formulae have been derived in healthy individuals.

Of the existing formulae tested, the James formula appeared to perform best in both the prediction of Ca^2+^ in the first cohort (r^2^ 0.271), and in the prediction of PTH in the second cohort in both hypocalcemic (r -0.11) and hypercalcemic patients (r +0.496), with similar performance to the newly derived formula (F1) incorporating all available biochemical parameters (F1) (r +0.499). Additionally, our study highlights that it may be necessary to derive bespoke reference ranges and formulae to adjust calcium in local centres to account for variations in assays used and population demographics, as well as to update these from time-to-time to take account of changes in performance of the assays (36, 37).

Strengths of the study include that this was a biochemistry laboratory centred audit enabling extraction of a large number of biochemical values analysed using the same analysers, however conversely a limitation is that granular clinical data on individual patient diagnoses/medications were not available. It is important to note that albumin measurements can differ depending on the assay used. For example, Roche BCP yields lower albumin readings than Abbott and differs from BCG and immunoturbidimetric methods. Consequently, it is important to note that older formulas such as Payne from 1973 (16) used a BCG method for albumin measurement. Additionally, due to Ca^2+^ being predominantly assessed in an acute setting, and PTH and vitamin D in the non-acute outpatient setting, it was not possible to directly assess both Ca^2+^ and PTH in the same cohort. Thus, we used the first cohort to derive a formula to estimate Ca^2+^, and then used a second cohort to correlate the estimated Ca^2+^ against PTH and vitamin D. Future prospective studies with concomitant measurement of Ca^2+^ and all other potentially relevant parameters e.g. FGF23 and patient histories in a controlled setting could enable more definitive exposition of the relationships between TCa and Ca^2+^ to enable more precise formulae to be derived. Additionally, urine calcium measures were not routinely available, to enable the exclusion of hypercalciuria as a cause of unexplained hyperparathyroidism.

## Conclusion

We find that adjustment of TCa for albumin does not outperform unadjusted values in the prediction of Ca^2+^ in an unrestricted dataset and that the relationship between PTH and calcium is strongest in the setting of hypercalcaemia. Of the existing formulae, the James formula performed best at predicting PTH levels in the setting of both high and low calcium levels.

## Data availability statement

The original contributions presented in the study are included in the article/**Supplementary Material**. Further inquiries can be directed to the corresponding author.

## Author contributions

MP wrote the original draft. MP and AC contributed equally as first authors to this manuscript. AS, ML, PE, SC, PM analysed the data. TT, JC, WD, AA edit, review and finalise the manuscript. All authors contributed to the article and approved the submitted version.

## Supplementary material

Supplementary Figure 1 Relationship between serum TCa and Ca^2+^ arranged by different variables. **(A)** Relationship between plasma TCa concentrations and Ca^2+^ arranged by different age categories. Values analysed by simple linear regression with r^2^ for <18 years =0.20 (n=267 samples), r^2^ for 18-49 years=0.23 (n=714 samples), r^2^ for 50-79 years=0.30 (n=1098 samples), r^2^ for >80 years=0.31 (n=543 samples). **(B)** Relationship between plasma TCa concentrations and Ca^2+^ arranged by different pH categories. Values analysed by simple linear regression with r^2^ for pH ≤ 7.12 = 0.21 (n=152 samples), r^2^ for 7.13-7.23 = 0.19 (n=258 samples), r^2^ for 7.24-7.34 = 0.22 (n=693 samples), r^2^ for 7.35-7.45 = 0.38 (n=1385 samples), r^2^ for ≥7.46 = 0.35 (n=2774 samples). **(C)** Relationship between plasma TCa concentrations and Ca^2+^ arranged by different creatinine categories. Values analysed by simple linear regression with r^2^ for <50μmol/L =0.367 (n=255 samples), r^2^ for 50-120μmol/L =0.258 (n=1900 samples), r^2^ for 121-1000μmol/L =0.310 (n=526 samples), r^2^ for 1001-2500μmol/L =0.0625 (n=24 samples). **(D)** Relationship between plasma TCa concentrations and Ca^2+^ arranged by different albumin categories. Values analysed by simple linear regression with r^2^ for 5-14g/L =0.0016 (n=30 samples), r^2^ for 15-24 g/L =0.253 (n=318 samples), r^2^ for 25-34 g/L =0.213 (n=1160 samples), r^2^ for 35-55 g/L =0.231 (n=1250 samples). **(E)** Relationship between plasma TCa concentrations and Ca^2+^ arranged by different globulin categories. Values analysed by simple linear regression with r^2^ for 5-14g/L =0.881 (n=10 samples), r^2^ for 15-24 g/L =0.227 (n=445 samples), r^2^ for 25-35 g/L =0.329 (n=1539 samples), r^2^ for 36-45 g/L =0.2560 (n=578 samples), r^2^ for 46-65 g/L = 0.132 (n=75 samples). **(F)** Relationship between plasma TCa concentrations and Ca^2+^ arranged by different total protein categories. Values analysed by simple linear regression with r^2^ for 20-40g/L =0.0047 (n=38 samples), r^2^ for 41-59 g/L =0.224 (n=806 samples), r^2^ for 60-78 g/L =0.256 (n=1635 samples), r^2^ for 79-100 g/L =0.176 (n=168 samples)

Supplementary Figure 2 Prediction of Ca^2+^ from routinely available variables in the restricted cohort. **(A)** Correlation matrix showing Spearman correlation coefficient of all available variables in the restricted (adult patients with normal pH (7.35-7.45), normal creatinine (<120 μmol/L), albumin (>15 g/L), total protein (>40 g/L) and calcium (2.2-2.6 mmol/L) levels) Ca^2+^ Prediction Cohort (n=537 samples). **(B)** Multivariable linear regression analysis of different combinations of variables for the prediction of ionized calcium in the restricted (adult patients with normal pH (7.35-7.45), normal creatinine (<120 μmol/L), albumin (>15 g/L), total protein (>40 g/L) and calcium (2.2-2.6 mmol/L) levels) Ca^2+^ Prediction Cohort (n=537 samples)
