# Supplementary figures and images for: Derivation and comparison of formulae for the adjustment of total calcium
Source: Front Endocrinol (Lausanne). 2023 May 12;14:1070443. doi: 10.3389/fendo.2023.1070443 (PMC10213740; doi:10.3389/fendo.2023.1070443)

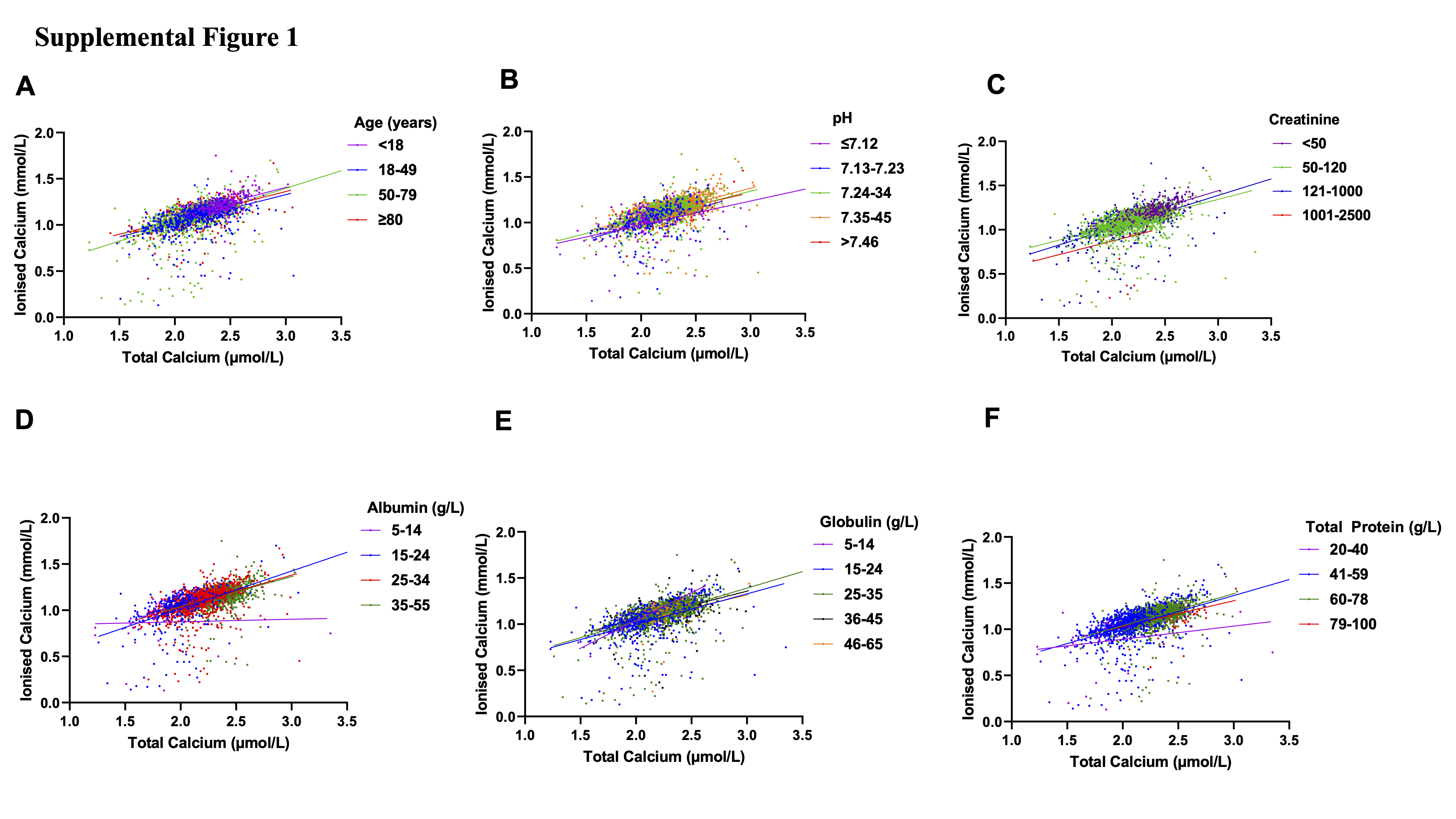

Supplement: Supplementary Figure 1 — Relationship between serum TCa and Ca2+ arranged by different variables. (A) Relationship between plasma TCa concentrations and Ca2+ arranged by different age categories. Values analysed by simple linear regression with r2 for <18 years =0.20 (n=267 samples), r2 for 18-49 years=0.23 (n=714 samples), r2 for 50-79 years=0.30 (n=1098 samples), r2 for >80 years=0.31 (n=543 samples). (B) Relationship between plasma TCa concentrations and Ca2+ arranged by different pH categories. Values analysed by simple linear regression with r2 for pH ≤ 7.12 = 0.21 (n=152 samples), r2 for 7.13-7.23 = 0.19 (n=258 samples), r2 for 7.24-7.34 = 0.22 (n=693 samples), r2 for 7.35-7.45 = 0.38 (n=1385 samples), r2 for ≥7.46 = 0.35 (n=2774 samples). (C) Relationship between plasma TCa concentrations and Ca2+ arranged by different creatinine categories. Values analysed by simple linear regression with r2 for <50μmol/L =0.367 (n=255 samples), r2 for 50-120μmol/L =0.258 (n=1900 samples), r2 for 121-1000μmol/L =0.310 (n=526 samples), r2 for 1001-2500μmol/L =0.0625 (n=24 samples). (D) Relationship between plasma TCa concentrations and Ca2+ arranged by different albumin categories. Values analysed by simple linear regression with r2 for 5-14g/L =0.0016 (n=30 samples), r2 for 15-24 g/L =0.253 (n=318 samples), r2 for 25-34 g/L =0.213 (n=1160 samples), r2 for 35-55 g/L =0.231 (n=1250 samples). (E) Relationship between plasma TCa concentrations and Ca2+ arranged by different globulin categories. Values analysed by simple linear regression with r2 for 5-14g/L =0.881 (n=10 samples), r2 for 15-24 g/L =0.227 (n=445 samples), r2 for 25-35 g/L =0.329 (n=1539 samples), r2 for 36-45 g/L =0.2560 (n=578 samples), r2 for 46-65 g/L = 0.132 (n=75 samples). (F) Relationship between plasma TCa concentrations and Ca2+ arranged by different total protein categories. Values analysed by simple linear regression with r2 for 20-40g/L =0.0047 (n=38 samples), r2 for 41-59 g/L =0.224 (n=806 samples), r2 for 60-78 g/L =0.256 (n= [file Image_1.jpg]

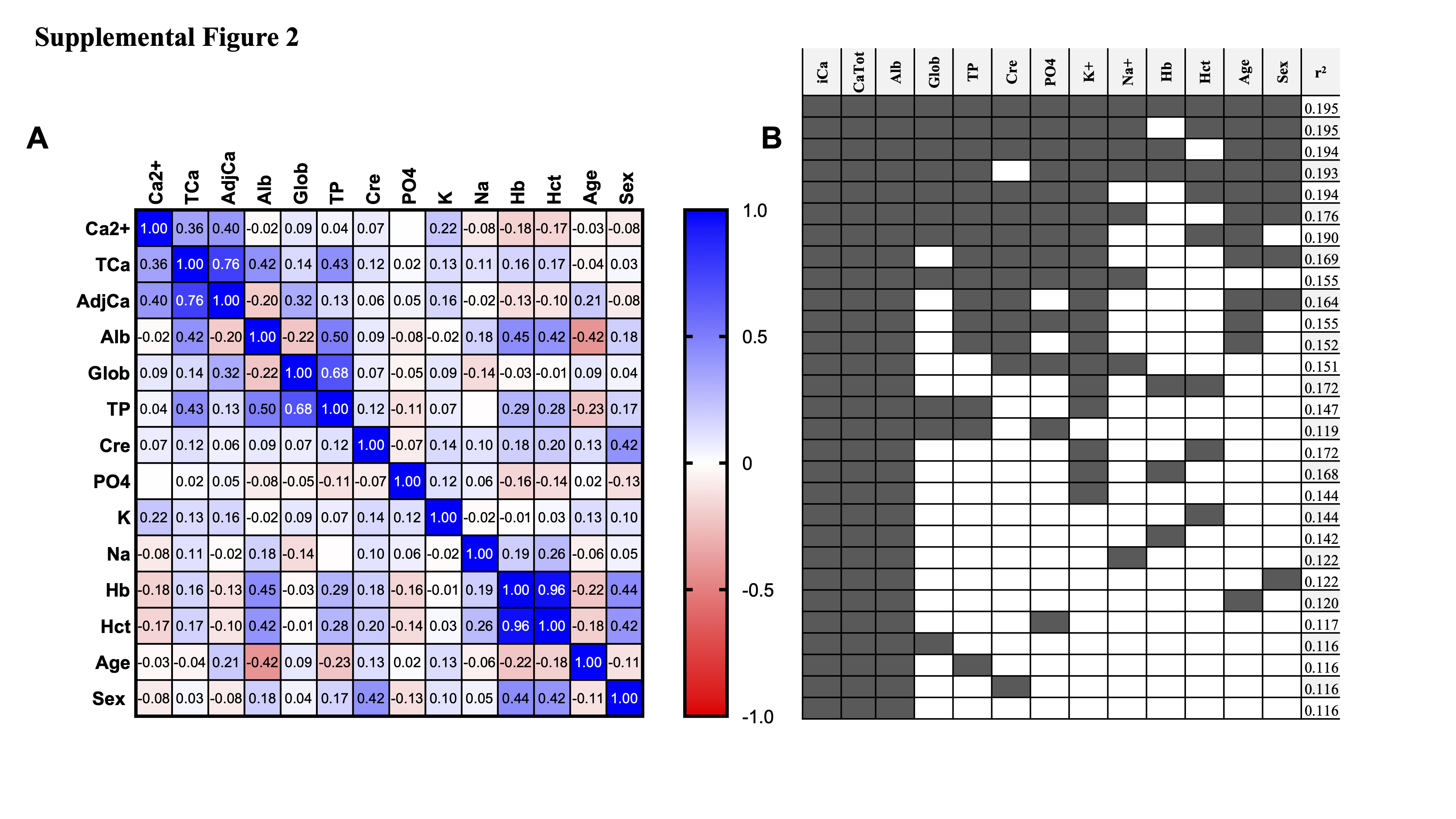

Supplement: Supplementary Figure 2 — Prediction of Ca2+ from routinely available variables in the restricted cohort. (A) Correlation matrix showing Spearman correlation coefficient of all available variables in the restricted (adult patients with normal pH (7.35-7.45), normal creatinine (<120 μmol/L), albumin (>15 g/L), total protein (>40 g/L) and calcium (2.2-2.6 mmol/L) levels) Ca2+ Prediction Cohort (n=537 samples). (B) Multivariable linear regression analysis of different combinations of variables for the prediction of ionized calcium in the restricted (adult patients with normal pH (7.35-7.45), normal creatinine (<120 μmol/L), albumin (>15 g/L), total protein (>40 g/L) and calcium (2.2-2.6 mmol/L) levels) Ca2+ Prediction Cohort (n=537 samples) [file Image_2.jpg]
